# Calmodulin-binding protein CBP60g functions as a negative regulator in *Arabidopsis* anthocyanin accumulation

**DOI:** 10.1371/journal.pone.0173129

**Published:** 2017-03-02

**Authors:** Bo Zou, Dongli Wan, Ruili Li, Xiaomin Han, Guojing Li, Ruigang Wang

**Affiliations:** 1 College of Life Sciences, Inner Mongolia Agricultural University, Hohhot, P. R. China; 2 Institute of Grassland Research, Chinese Academy of Agricultural Sciences, Hohhot, P. R. China; 3 Wulanchabu Center for Disease Control and Prevention, Jining, P. R. China; 4 Department of Histology and Embryology, Baotou Medical College, Baotou, P. R. China; National Taiwan University, TAIWAN

## Abstract

Anthocyanins, a kind of flavonoid, normally accumulate in the flowers and fruits and make them colorful. Anthocyanin accumulation is regulated via the different temporal and spatial expression of anthocyanin regulatory and biosynthetic genes. CBP60g, a calmodulin binding protein, has previously been shown to have a role in pathogen resistance, drought tolerance and ABA sensitivity. In this study, we found that CBP60g repressed anthocyanin accumulation induced by drought, sucrose and kinetin. The expression pattern of *CBP60g* was in accordance with the anthocyanin accumulation tissues. Real-time qPCR analysis revealed that the anthocyanin biosynthetic genes *CHS*, *CHI* and *DFR*, as well as two members of MBW complex, *PAP1*, a MYB transcription factor, and *TT8*, a bHLH transcription factor, were down regulated by CBP60g.

## Introduction

Anthocyanins, a kind of natural hydrosoluble pigment produced by the flavonoid biosynthetic pathway, extensively exists in all kinds of plants and color the petals and fruits. Plant benefits from these pretty colors to attract the insects to pollinate and transmit the seeds [1]. In vegetative tissues, anthocyanins also have photoprotective roles by providing a light-absorbing screen for photosynthetic cells [2, 3], and serve as scavenger to remove reactive oxygen species under light stress conditions [4]. To fulfill these functions, anthocyanin biosynthesis deserves an accurate regulation in plant.

Anthocyanin biosynthetic and regulatory genes show temporal and spatial expression pattern differently and this enable the fine control of anthocyanin accumulation [1, 5, 6]. The understanding in flavonoid biosynthetic pathway and its regulatory components is increasing [1]. The early biosynthetic genes (EBGs) include *CHS* (chalcone synthase), *CHI* (chalcone isomerase), *F3H* (favone 3-hydroxylase), and *FLSM* (favonol synthase). The late biosynthesis genes (LBGs) include *DFR* (dihydrofavonol 4-reductase), *LDOX* (leuco anthocyanidin dioxygenase), and *UF3GT* (UDP-glucose:favonoid 3-Oglucosyl transferase) and so on [7]. Many anthocyanin biosynthetic genes were regulated by MBW (MYB-bHLH-WD40) protein complex consisting of R2R3-MYB, bHLH and a WD-repeat containing protein [8, 9]. R2R3-MYBs, owing to their multiple expression pattern compared with bHLH and WD40, are the essential regulatory components in the complex. [2, 10, 11].

PAP1 (PRODUCTION OF ANTHOCYANIN PIGMENT 1), a R2R3-MYB transcription factor, can interact with a bHLH transcription factor TT8 (transparent test 8), EGL3 (enhancer of glabra3) or GL3 (glabra3), and a WD-repeat transcription factor TTG1 (transparent testa 1), and these ternary complexes regulate anthocyanin synthesis [12]. The *pap1* knockout mutant showed a less anthocyanin accumulation phenotype than wild-type, while overexpression of *PAP1* increased anthocyanin accumulation [11, 13].

The accumulation of *PAP1* transcripts is regulated by temperature, concentration of sucrose, hormone treatment, strength and wavelength of light [14]. Cytokinin positively regulated anthocyanin accumulation caused by sucrose, and this process is PAP1 mediated [15, 16]. Ethylene has been proved to play a negative role in anthocyanin accumulation [14, 17]. However, the exact regulatory components upstream PAP1 are not clear.

CBP60s are plant specific calmodulin-binding proteins first identified in maize [18–20]. In *Arabidopsis thaliana*, there are eight CBP60s, named CBP60a-g, and SARD1 [21]. Five members of CBP60s can interact with CaM (calmodulin) in a Ca^2+^ dependent manner following treatment with elicitors [21]. CBP60g lacks the CaM interaction C-terminal, with a CaM interaction N-terminal instead [22]. Previous studies have shown that most of the members of CBP60 family play roles in pathogen and drought resistance [18, 23, 24]. CBP60g acts synergistically with SARD1 in the pathogen resistance, while antagonistically with CBP60a [18, 23–25].

Previous studies focused on the role of CBP60g in the pathogen resistance. Wang et al. revealed that CBP60g participated in SA (salicylic acid) synthesis and contributed to pathogen resistance [22]. The *cbp60g* mutant was found to support more bacterium growth than the wild-type in a bacterium growth assay[24]. Zhang et al. further confirmed that SARD1 (Systemic Acquired Resistance Deficient 1) and CBP60g regulated the SA biosynthetic gene, *ICS1* (Isochorismate synthase 1), while the induction of *ICS1* was blocked in the *cbp60g* mutant. CBP60g fulfilled this role by binding to the promoter of *ICS1* and functioning as a transcription activator [26]. Wan et al. found that *CBP60g* overexpression plants accumulated more *ICS1* transcripts and SA, and were more resistant to pathogen [23].

In addition to its role in pathogen resistance, Wan et al. indicated that CBP60g was also involved in drought tolerance and ABA sensitivity. In this paper, we found that CBP60g could regulate the expression of two members of MBW complex, PAP1, a MYB transcription factor, and TT8, a bHLH transcription factor, thus control the anthocyanin synthesis, and for the first time linked calcium signaling to the anthocyanin accumulation.

## Materials and methods

### Plant materials and growth conditions

*Arabidopsis thaliana* wild-type was Columbia-0, the *cbp60g* mutant and *CBP60g* overexpression lines were also in the Columbia background. The T-DNA insertion allele of *CBP60g* (*cbp60g-1*; At5g26920; SALK-023199) was obtained from the *Arabidopsis* Biological Resource Center (ABRC).

Seeds were surface sterilized by sequentially immersed in 75% ethanol or 100% ethanol with 0.05% Tween-20 for 10 min each. After 3 days stratification at 4°C on half strength MS medium, plants were set into 22°C growth chamber with a 16h/8h of light/dark cycle. After 10 days, the seedlings were transferred to a 1:1 mixture of peat soil and vermiculite in the same growth chamber. Swimming plants were growth in GC (gas chromatography) vial and performed as previously reported [23].

### Drought treatment

We use two or three-weeks-old plants to observe the anthocyanin accumulation under drought treatment. Either 4 three-weeks-old plants or 200 two-weeks-old seedlings grown in a pot with 80g mixture soil were undergoing a water limitation, that 30 mL water each pot was supplied every 3 days. Six biological replicates were performed.

### Measurement of anthocyanin content

About 0.1g samples were grounded in 1.5 mL Eppentdorf tube and 1mL methanol contain 1% HCl was added. After centrifugation at 13000 rpm for 20 min, the absorbance of supernatants were measured at 528 nm and 657 nm using Beckman DU800 (USA). The content of anthocyanin was quantified using the formula A530-1/4(A657) to compensate for the contribution of chlorophylls. Three biological replicates were performed.

### Anthocyanin Induced Condition (AIC)

About 100 seeds were sown in half strength liquid MS medium with 3% sucrose. After 12 days, anthocyanin accumulation can be observed. To observe anthocyanin among different lines in the same plate, we use half strength solid MS medium contain 3% sucrose, 40 μM kinetin or 7% sucrose as AIC. Each plate contains 30 seedlings for one line, and observed 14 days after treatment. Three biological replicates were performed.

### Histochemical GUS assay

Pro_CBP60g_::GUS transgenic plants generated by previously study [23] were grown in half strength liquid MS medium with 3% sucrose for 14 days. GUS staining was performed as previously described [23]. Samples were immersed in staining buffer (50 mM Na_2_HPO_4_–NaH_2_PO_4_, pH 7.3, 2 mM K_4_Fe(CN)_6_, 2 mM K_3_Fe(CN)_6_, 0.1% Triton X-100) with 0.5 mg mL^-1^ X-Gluc and incubated in 37°C for 6–12h. After decolorized by ethanol, the GUS staining patterns were dphotographe under a dissecting microscope (Nikon SMZ800).

### Kinetin and sucrose treatment

Kinetin (KT) and sucrose were used to induce the expression of anthocyanin synthesis related genes. Twelve -day-old seedlings growing in 20 mL GC vials were treated with a final concentration of 100μM KT or 150 mM (5.13%) of sucrose. Samples were harvested at various time points.

### Quantitative real-time PCR analysis

Total RNA was extracted from 30 mg samples. After DNase I (Ambion Cat# AM2224) treatment, 500 ng total RNA was used for reverse transcription using M-MLV reverse transcriptase kit (TaKaRa). After 40-fold dilution, 5 μL cDNA was used as the template of real-time PCR using SYBR Premix Ex Taq (TaKaRa), with a Roche LightCycler 480. The PCR was performed with the following cycling profile: 95°C for 1 min; 40 cycles at 95°C for 5 s, 60°C for 30 s, and 72°C for 10 s. *AtEF1α* was used as reference gene. Three technical repetitions were performed for each experiment, and at least three independent biological replicates were performed. The primers used for qRT-PCR were listed in S1 Table.

### EGTA treatment

Ethyleneglycol-bis(beta-aminoethylether)-N,N'-tetraacetic acid (EGTA) was added into half strength MS medium contain 40μM KT and 3% sucrose to chelate the Ca^2+^. The 18-day-old seedlings were treated and observed.

## Results

### CBP60g affected the anthocyanin accumulation during drought stress

**Fig 1:**
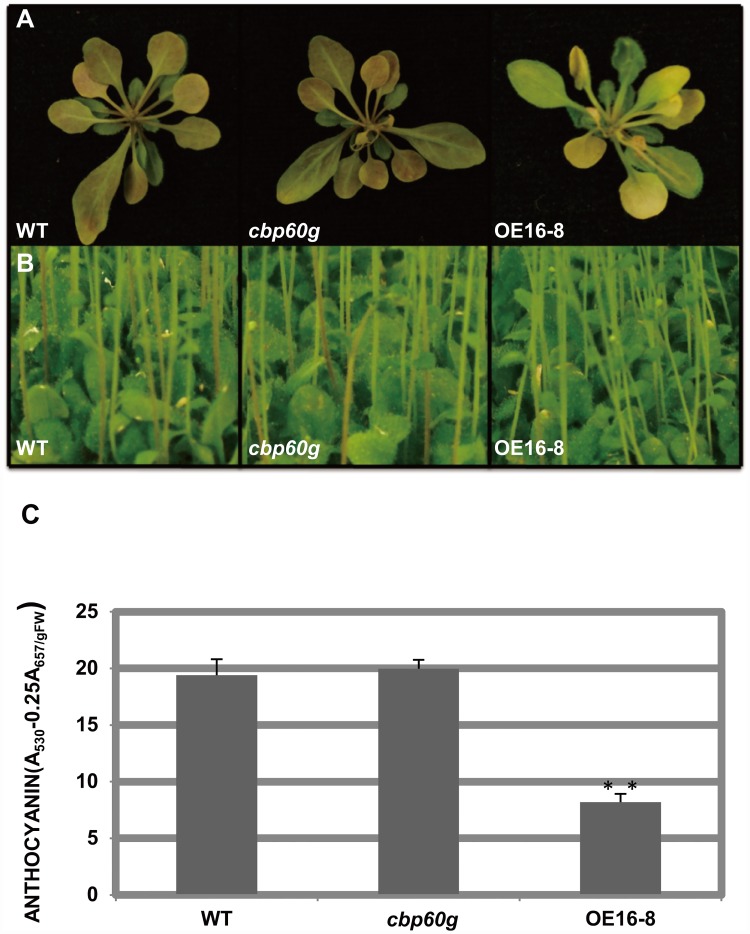
Anthocyanin accumulation under drought treatment was affected by CBP60g. Anthocyanin accumulation of three-week-old (A) or two-week-old (B) plants was observed among wild-type, the *cbp60g* mutant and *CBP60g* overexpression lines under water limitation condition. Water was supplied 30 mL per pot containing 80g mixture soil every three days. (C) The content of anthocyanin in (A) was quantified. Three biological replicates were performed for each experiment. Each data point represents the average of three technical replicates ±SD.

Previously, it was reported that CBP60g play an important role in *Arabidopsis* disease resistance [18, 23, 24]. And *CBP60g* overexpression lines exhibited a drought-tolerance phenotype [22]. To further investigate the mechanism of CBP60g in plants drought tolerance, different genotypes of plants were exposed to drought stress by limiting the water supply for two weeks, anthocyanin did not accumulate as much in the *CBP60g* overexpression line (OE16-8) as in wild-type (Columbia-0) plants, especially in the abaxial side of the rosette leaves and the stems (Fig 1A and 1B). Consistent with this, measurement of anthocyanin content of the rosette leaves indicated that it was three times less in *CBP60g* overexpression line than that in wild-type (Fig 1C). This suggests that CBP60g suppresses the anthocyanin accumulation under drought stress.

### CBP60g as a negative regulator in sucrose and kinetin induced anthocyanin accumulation

As the mechanism of anthocyanin accumulation under drought stress is not clear, in this study, we used sucrose and kinetin containing medium as anthocyanin inductive conditions (AIC) as previously described [27]. Accumulating data revealed that sucrose containing medium induced anthocyanin synthesis either independently or synergistically with cytokinin. The presence of sucrose appears to promote anthocyanin accumulation via controlling the expression of *PAP1*, which is a component of the MBW (MYB-bHLH-WD40) and regulate the expression of anthocyanin synthase [16, 28–30].

**Fig 2:**
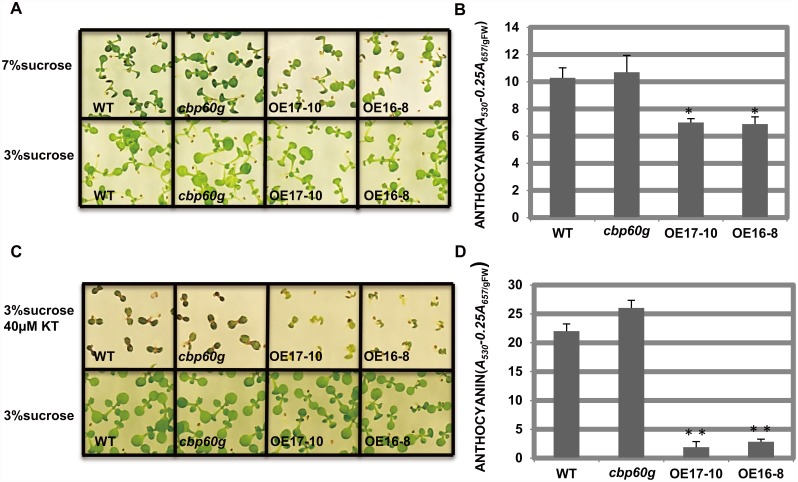
CBP60g suppressed anthocyanin accumulation induced by sucrose and kinetin. Wild-type, the *cbp60g* mutant and *CBP60g* overexpression lines grown in the solid 1/2 MS medium contain 7% sucrose (A) or in the solid 1/2 MS medium contain 3% sucrose and 40 μM kinetin (C) as anthocyanin induced condition (AIC) were observed. The photograph was taken after 14 days of growth. (B) and (D) The anthocyanin content was quantified in (A) and (C). Three biological replicates were performed for every experiment. Each data point represents the average of three technical replicates ±SD.

Seedling of the *cbp60g* mutant, *CBP60g* overexpression line and WT was growing in solid half strength MS medium containing either 3% sucrose or 7% sucrose for 14 days, OE16-8 plants accumulated less anthocyanin than wild-type, while the *cbp60g* mutantaccumulated more anthocyanin than wild-type in 7% sucrose containing medium (Fig 2A and 2B). Furthermore, in 3% sucrose and 40 μM kinetin containing solid half strength MS medium (AIC), the accumulation of anthocyanin is higher in the *cbp60g* mutant and lower in the *CBP60g* overexpression lines compared with WT (Fig 2C and 2D). In addition, the *CBP60g* overexpression lines showed greater growth inhibition than the wild-type and the *cbp60g* mutant when the concentration of kinetin was increased to 100 μM (Fig 2C and S1 Fig).

Taken together, these results indicated that CBP60g was a negative regulator in anthocyanin accumulation.

### The expression pattern of CBP60g was coincident with anthocyanin accumulation pattern

**Fig 3:**
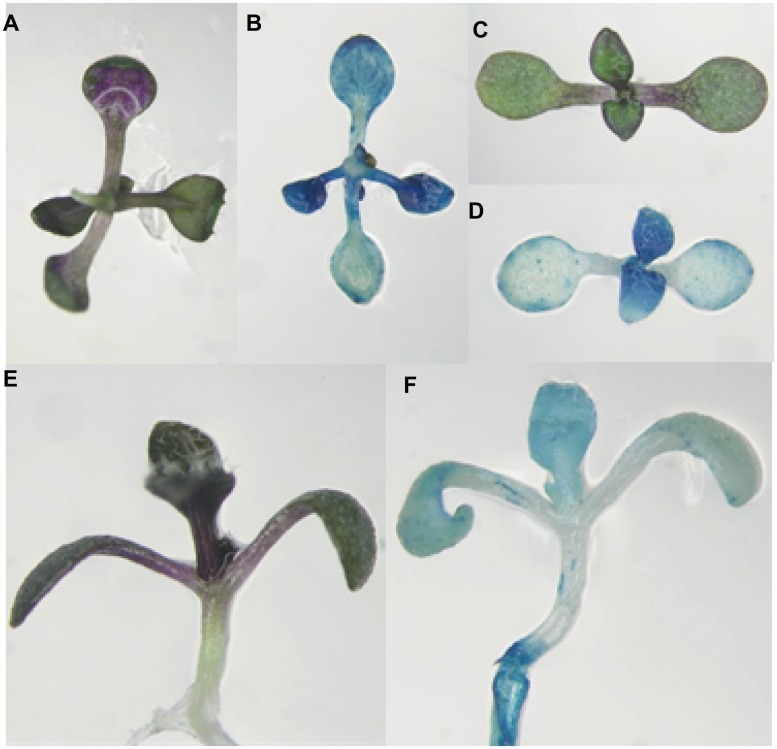
The comparison of the anthocyanin accumulation tissues and the CBP60g expression pattern. (A), (C) and (E) The anthocyanin accumulation tissues. (B), (D) and (F) CBP60g expression tissues. (A) and (B) The abaxial side of 14 days seedlings. (C) and (D) The adaxial side of 14 days seedlings. (E) and (F) The flank side of 14 days seedlings.

Our previous study has shown that *CBP60g* is expressed in various tissues and organs [23]. In this study, we used the Pro_CBP60g_::GUS transgenic plants [23] grown in the liquid half strength MS medium with 3% sucrose to observe the histochemical localization of GUS activity, along with anthocyanin accumulation. Anthocyanin accumulated in the abaxial side of leaves, especially in true leaves (Fig 3A and 3B). While in the adaxial side, anthocyanin accumulated in cotyledons and the edge of the true leaves (Fig 3C and 3D). In the flank side, we also observed that anthocyanin accumulated in cotyledons and true leaves. GUS activity was also detected in the same tissues Fig 3E and 3F). We also found that *CBP60g* itself was responsive to kinetin (S2A Fig).

### The anthocyanin biosynthetic and regulatory genes were negatively regulated by CBP60g

**Fig 4:**
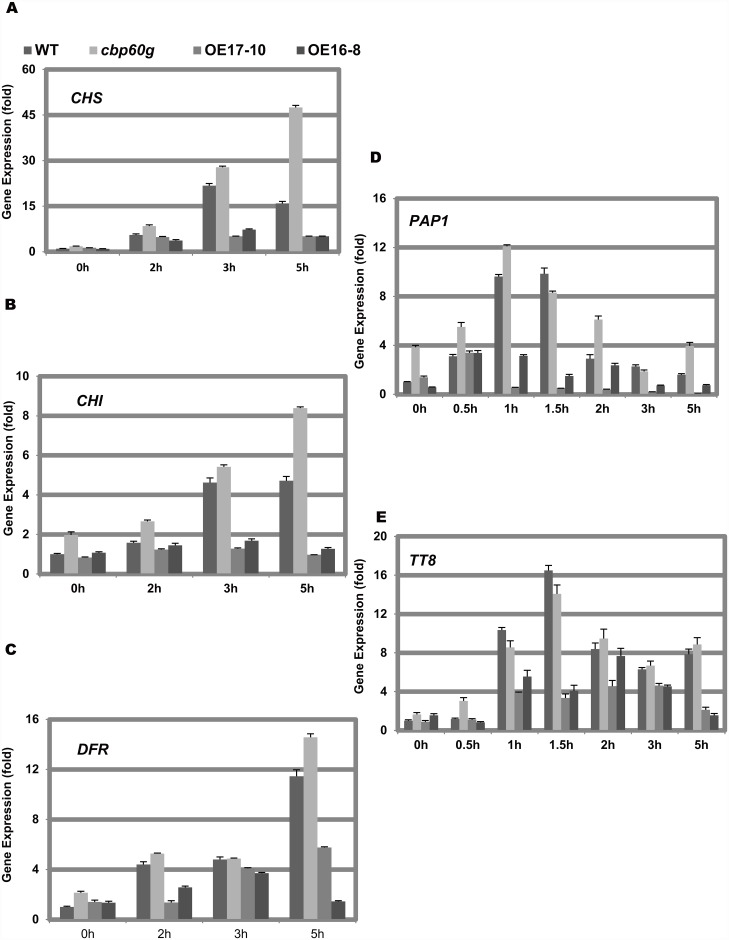
CBP60g repressed the expression of anthocyanin biosynthetic and regulatory genes under AIC. Twelve-day-old wild-type, the *cbp60g* mutant and *CBP60g* overexpression lines grown in the liquid 1/2 MS medium were treated with 100 μM kinetin. The expression of anthocyanin biosynthetic genes *CHS*, *CHI* and *DFR*, along with the anthocyanin regulatory genes *PAP1* and *TT8* were detected. Three biological replicates were performed for every experiment. Each data point represents the average of three technical replicates ±SD.

It has been pointed out that the anthocyanin biosynthetic genes are induced under sucrose and cytokinin treatment [31, 32]. We detected the anthocyanin regulatory genes and biosynthetic genes among the wild-type, the *cbp60g* mutant and the *CBP60g* overexpression line under sucrose and cytokinin treatment using qRT-PCR. *ARR5* was used as a marker gene to validate the treatment (S3 Fig). The induction of biosynthetic genes *CHS*, *CHI* and *DFR* in the *cbp60g* mutant was more intensive than that in wild-type, however, it was reduced in the *CBP60g* overexpression line Fig 4A, 4B and 4C). The induction of anthocyanin regulatory genes such as *PAP1* and *TT8* (belonging to the MBW) was similar to the synthetic genes (Fig 4D and 4E). The induction of *PAP2*, *TT2*, *EGL2*, *MYBL2* and *GL3*, which also belongs to the MBW complex, were not affected among different genotypes (S3 Fig). The result indicated that CBP60g negatively regulated the anthocyanin synthesis through PAP1 and TT8. We also checked the expression of *CHS*, *CHI*, *DFR*, *PAP1* and *TT8* under 150 mM sucrose treatment, and confirmed that CBP60g suppressed the expression of these genes. (S4 Fig).

### Anthocyanin accumulation regulated by CBP60g was calcium-independent

**Fig 5:**
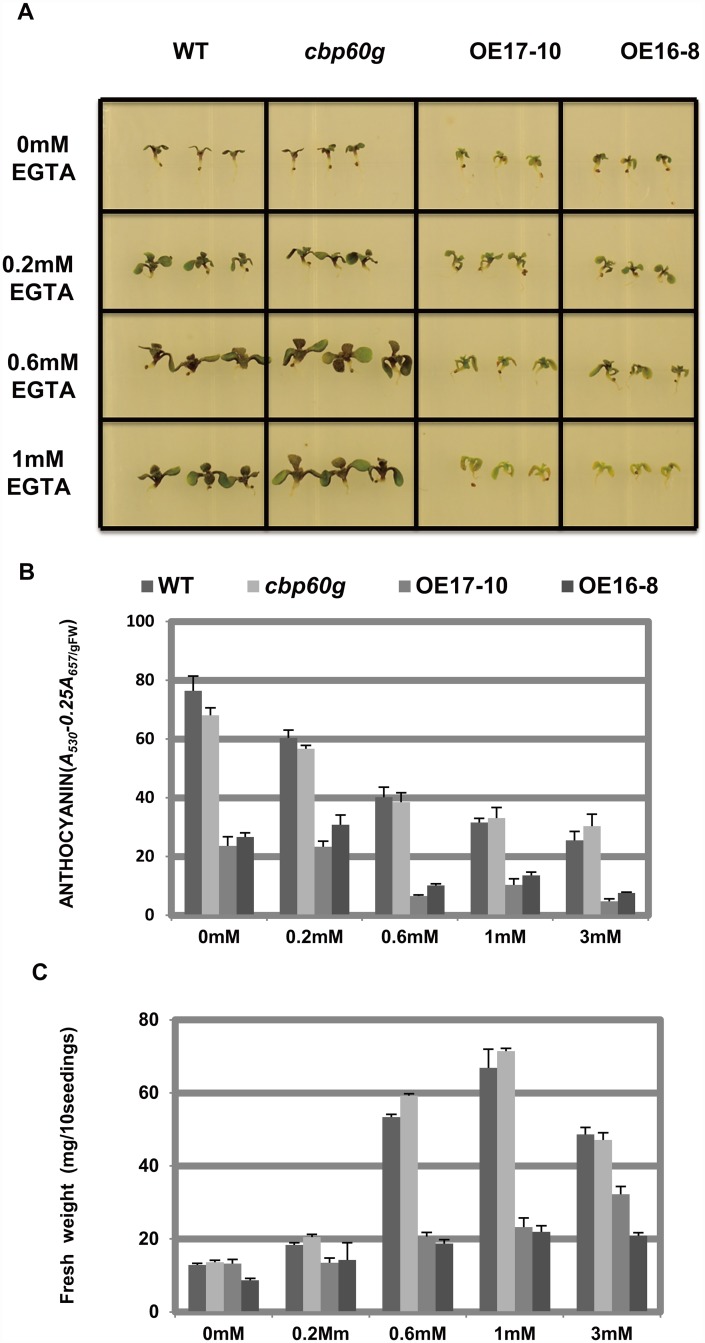
CBP60g repressed the anthocyanin accumulation in a calcium independent manner. Wild-type, the *cbp60g* mutant and *CBP60g* overexpression lines grown in the solid 1/2 MS medium with 3% sucrose, 40 μM kinetin and various concentrations of EGTA were observed (A). The fresh weight and anthocyanin content were quantified (B) and (C). Three biological replicates were performed for every experiment. Each data point represents the average of three technical replicates ±SD.

CBP60g, as a plant specific calmodulin binding protein, has the innate relationship with Ca^2+^ [23]. We had known that the expression of *CBP60g* is induced by Ca^2+^ (S5 Fig). Then we try to investigate whether Ca^2+^ can influence the repression of anthocyanin synthesis caused by CBP60g. Ethyleneglycol-bis(beta-aminoethylether)-N,N'-tetraacetic acid (EGTA) was used to chelate Ca^2+^ in the AIC medium. Wild-type, the *cbp60g* mutant and the *CBP60g* overexpression lines grown in the solid half strength MS medium supplemented with 3% sucrose, 40 μM kinetin and various concentrations of EGTA were observed (Fig 5A). We found that anthocyanin accumulation was weakened along with the decrease of Ca^2+^ (Fig 5B). The extent of reduction in anthocyanin accumulation was similar among wild-type, the *cbp60g* mutant and CBP60g overexpression lines. This result showed that CBP60g regulated anthocyanin accumulation is calcium independent.

Interestingly, the growth retardation of wild-type and the *cbp60g* mutant caused by kinetin was greatly reduced by the reduction of Ca^2+^, however, *CBP60g* overexpression lines did not appear to recover in any concentration of EGTA (Fig 5C).

## Discussion

### 1 CBP60g function as feedback repressor in Ca^2+^ promoted anthocyanin accumulation

The role of calcium signaling in anthocyanin accumulation has been reported recently. Shin et al. discovered that Ca^2+^ induced Arabidopsis anthocyanin accumulation, it was depressed while Ca^2+^ was inactivated by adding EGTA into medium [17]. Peng et al. clarified that *Fragaria vesca* UDP-glucosyltransferase (FvUGT1) activity was inhibited by anthocyanidin, and was alleviated via calmodulin binding to FvUGT1 [33]. CDPKs, as a part of calcium signaling, have an important role in plant growth and stress response. CDPKs regulate the activity of transcription factors and metabolic enzymes via the molecular interaction [34]. Schulz et al. revealed that PARP suppressed anthocyanin accumulation via controlling the anthocyanin related transcription factors expression, and this is similar to the role of CBP60g, one of the transcription factors in anthocyanin accumulation [35]. The anthocyanin synthesis in *Medicago truncatula* was negatively controlled by MYB2, which itself was regulated by CBP60g[23, 36]. In our study, anthocyanin content decreased in Arabidopsis grown in medium containing EGTA (Fig 5), which was in accordance with the previous studies that calcium induced anthocyanin accumulation,. However, it increased when grown in medium with extra Ca^2+^ (S5 Fig). In brief, calcium induces anthocyanin accumulation and the expression of *CBP60g*. However, CBP60g represses this anthocyanin accumulation conversely. An explanation is that CBP60g mediates a negative feedback, which suppresses anthocyanin accumulation (illustrated in S6 Fig). After stress treatment and signaling activation, intercellular calcium concentration increases which stimulates anthocyanin accumulation in turn and the expression of *CBP60g*. Then, CBP60g induces the expression of *PAP1* and *TT8* which are components of the MBW complex. However, this interaction is not quite clear so far. Finally, the anthocyanin synthetic genes are repressed and anthocyanin accumulation is blocked. It is without doubt that CBP60g, as a transcription factor, regulates anthocyanin accumulation in a currently unknown manner, perhaps by regulating anthocyanin synthesis related genes and competitively inhibiting the calmodulin function. The negative feedback appears to be calcium independent (Fig 5).

### 2 How do CBP60g regulate the anthocyanin biosynthetic or regulatory genes

Plant anthocyanin synthesis is regulated by the MBW complex. MYB transcription factor was a key component in the MBW complex [11, 16]. PAP1, a MYB2 transcription factor, plays an important role in sucrose induced anthocyanin accumulation [11, 37]. MBW complex regulates the expression of many anthocyanin synthesis genes. Our results show that CBP60g also regulates the expression of many anthocyanin synthesis related genes, such as *PAP1*, *TT8*, *CHS*, *CHI* and *DFR* (Fig 4 and S4 Fig). Zhang et al. revealed that CBP60g regulates the expression of *ICS1* via directly binding to the GAAATTT motif of its promoter[26]. We searched the promoters from the 2000bp upstream to 200bp downstream of the start codon of *PAP1*, *TT8*, *CHS*, *CHI* and *DFR* for the CBP60g binding motif and found it is only present in the promoter of *CHS* and *PAP1*. This suggests that CBP60g may regulate other genes in an indirect way, such as via MYB2.

### CBP60g blocked calcium regulated growth inhibition caused by kinetin

The crosstalk between cytokinin and calcium signaling pathway has been discovered. Peng et al (1996) has revealed that Ca^2+^ inhibits senescence-retarding effect of cytokinins in detached rice leaves [38]. Calcium antagonists and calmodulin inhibitors repress cytokinin-induced bud formation in Funaria [39]. In this study, we also found that EGTA, as a calcium chelator, could block the growth retardation caused by kinetin (Fig 5).

The reduced growth phenotype caused by kinetin was not removed by decreasing Ca^2+^ in the *CBP60g* overexpression lines. This indicated that CBP60g participated in cytokinin signaling pathway, and played a role in the crosstalk between cytokinin and Ca^2+^.

## Supporting information

S1 Fig The *CBP60g* overexpression plants were lethal under 100μM kinetin treatment. Plants were vertically grown on the 1/2 MS medium with 100μM kinetin and photographed 14 days after treatment.
(TIF)

S2 Fig *CBP60g* and *ARR5* were induced by kinetin treatment. Twelve-day-old wild-type, the *cbp60g* mutant and *CBP60g* overexpression lines grown in the liquid 1/2 MS medium were treated with 100 μM kinetin kinetin. Three biological replicates were performed for every experiment. Each data point represents the average of three technical replicates ±SD.
(TIF)

S3 Fig The expression of *PAP2*, *TT2*, *EGL2*, *MYBL2* and *GL3*. Twelve-day-old wild-type, the *cbp60g* mutant and the *CBP60g* overexpression lines grown in the liquid 1/2 MS medium were treated with 100 μM kinetin kinetin. Three biological replicates were performed for every experiment. Each data point represents the average of three technical replicates ±SD.
(TIF)

S4 Fig The expression of *PAP1*, *TT8*, *DFR*, *CHI* and *CHS* under sucrose treatment. Twelve-day-old wild-type, the *cbp60g* mutant and *CBP60g* overexpression lines grown in the liquid 1/2 MS medium were treated with 150 Mm sucrose. Three biological replicates were performed for every experiment. Each data point represents the average of three technical replicates ±SD.
(TIF)

S5 Fig Calcium induced anthocyanin accumulation. (A) Twelve-day-old wild-type, the *cbp60g* mutant and *CBP60g* overexpression lines grown in the solid 1/2 MS medium with 3% sucrose and 100 mM CaCl_2_ were observed. (B) The expression of *CBP60g* was induced under 50 mM CaCl_2_.
(TIF)

S6 Fig CBP60g suppresses Arabidopsis anthocyanin accumulation. Light and sucrose induce anthocyanin accumulation, cytokinin and calcium signaling participate in this process. Calcium signaling increases the anthocyanin accumulation, while on the other hand, induces *CBP60g* expression thus represses the anthocyanin accumulation. These form a negative feedback. We suggest that CBP60g regulates anthocyanin accumulation through PAP1 and TT8, which are the components of MBW complex.
(TIF)

S1 Table Primers used for qRT-PCR analysis in this work. (DOC)
