# Supplementary figures and images for: Calmodulin-binding protein CBP60g functions as a negative regulator in Arabidopsis anthocyanin accumulation
Source: PLoS One. 2017 Mar 2;12(3):e0173129. doi: 10.1371/journal.pone.0173129 (PMC5333885; doi:10.1371/journal.pone.0173129)

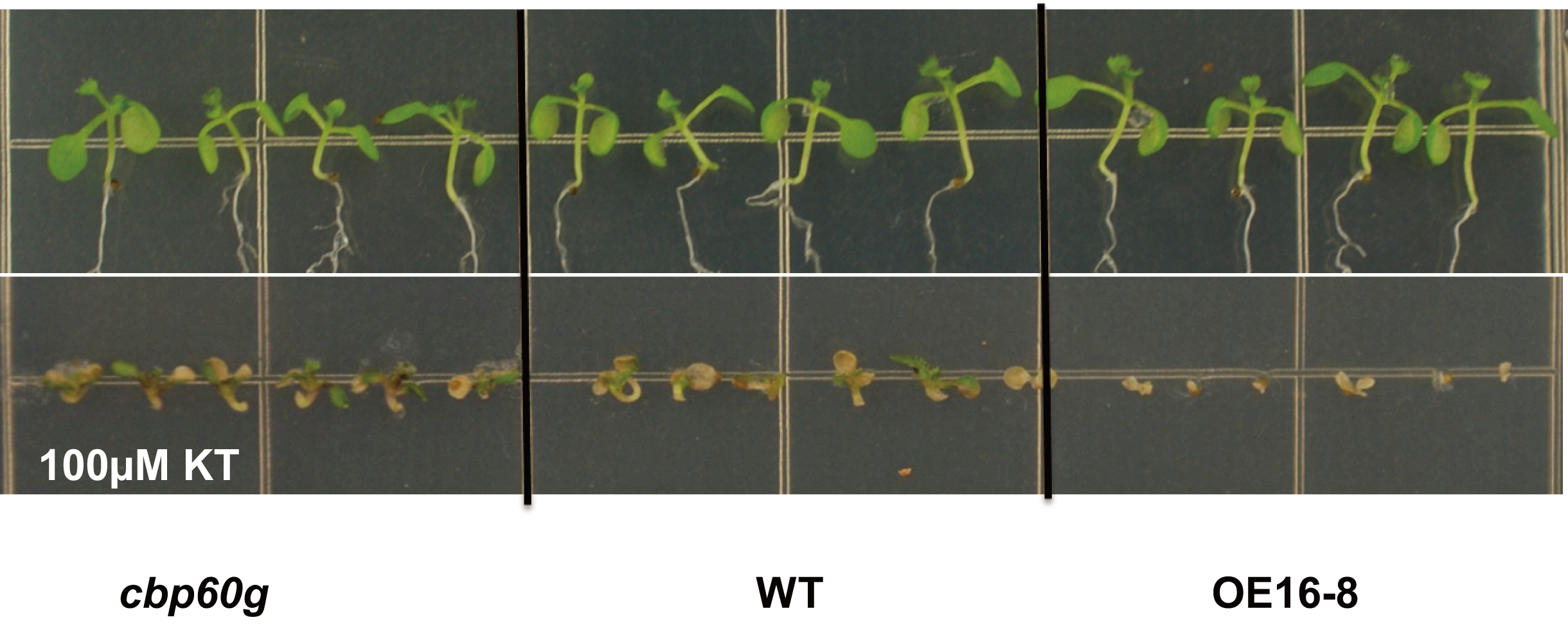

Supplement: S1 Fig — Plants were vertically grown on the 1/2 MS medium with 100μM kinetin and photographed 14 days after treatment. (TIF) [file pone.0173129.s001.tif]

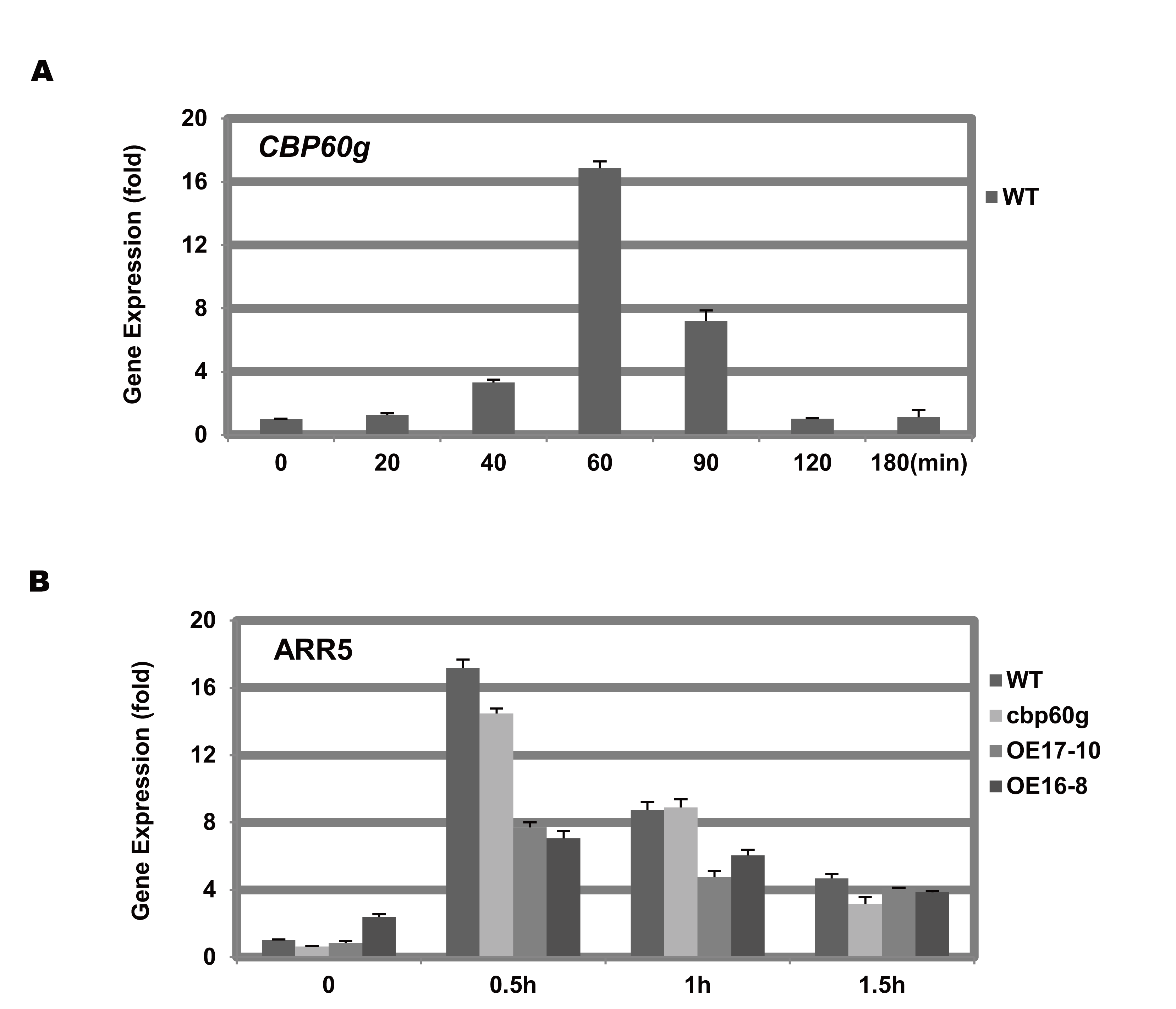

Supplement: S2 Fig — Twelve-day-old wild-type, the cbp60g mutant and CBP60g overexpression lines grown in the liquid 1/2 MS medium were treated with 100 μM kinetin kinetin. Three biological replicates were performed for every experiment. Each data point represents the average of three technical replicates ±SD. (TIF) [file pone.0173129.s002.tif]

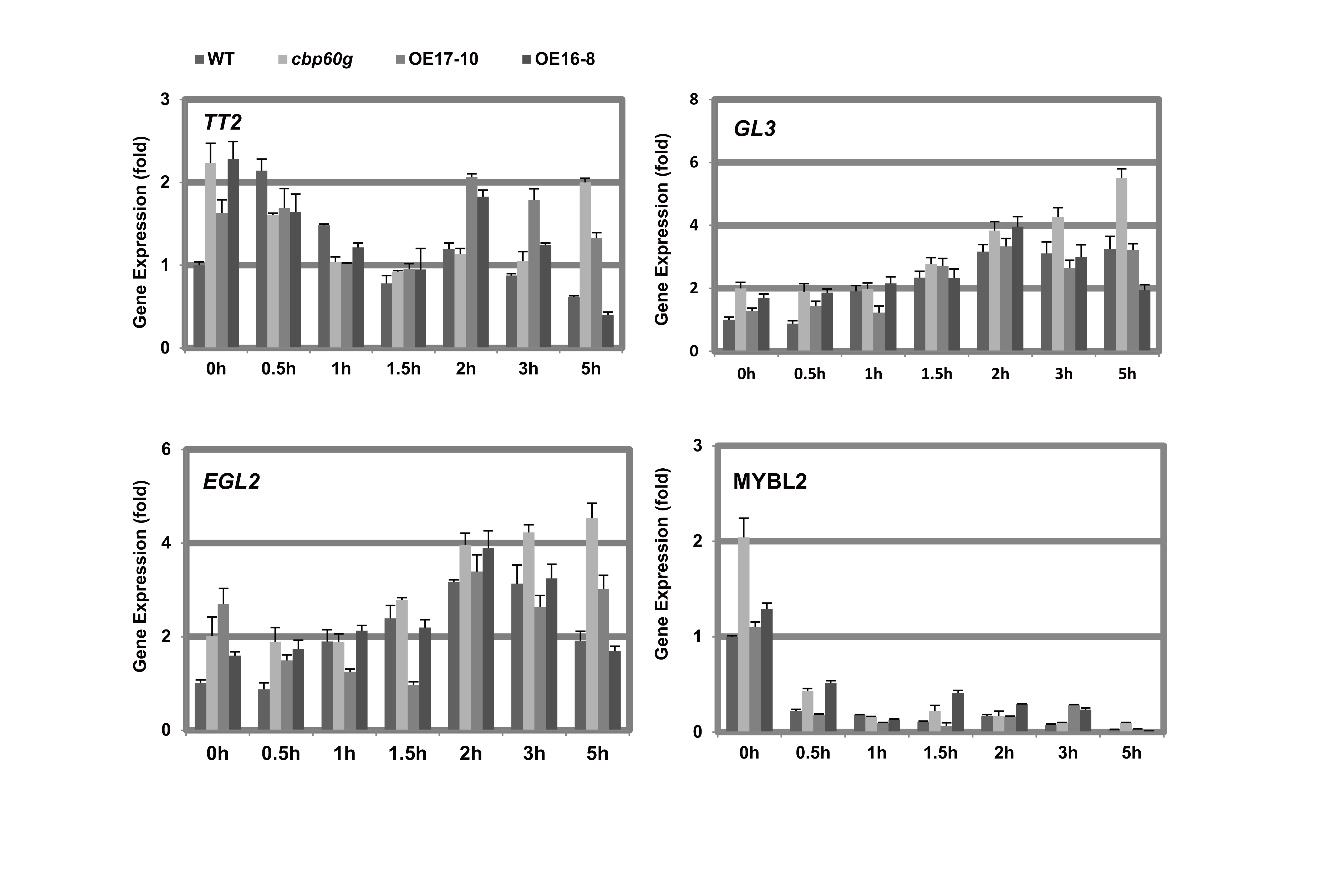

Supplement: S3 Fig — Twelve-day-old wild-type, the cbp60g mutant and the CBP60g overexpression lines grown in the liquid 1/2 MS medium were treated with 100 μM kinetin kinetin. Three biological replicates were performed for every experiment. Each data point represents the average of three technical replicates ±SD. (TIF) [file pone.0173129.s003.tif]

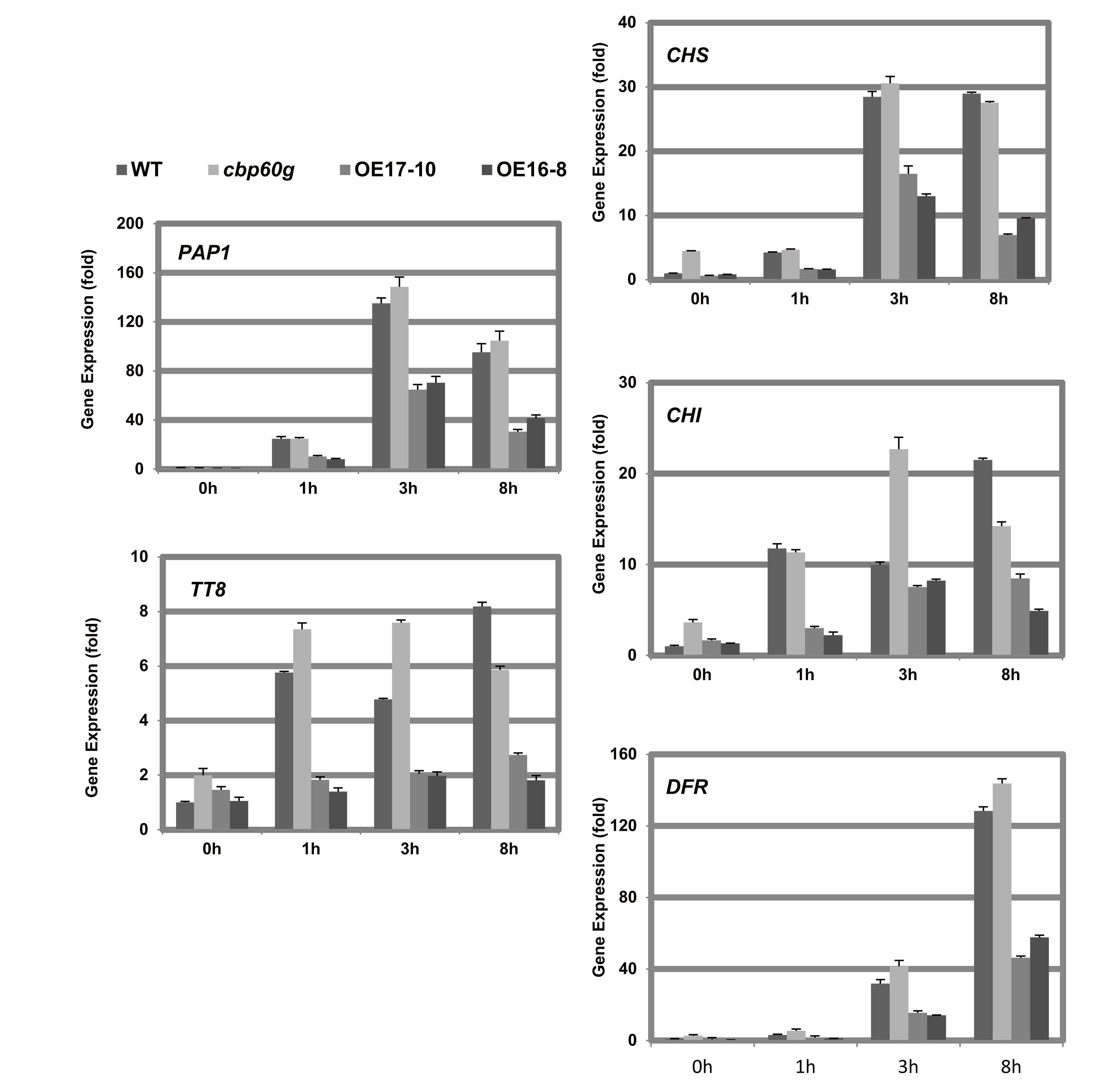

Supplement: S4 Fig — Twelve-day-old wild-type, the cbp60g mutant and CBP60g overexpression lines grown in the liquid 1/2 MS medium were treated with 150 Mm sucrose. Three biological replicates were performed for every experiment. Each data point represents the average of three technical replicates ±SD. (TIF) [file pone.0173129.s004.tif]

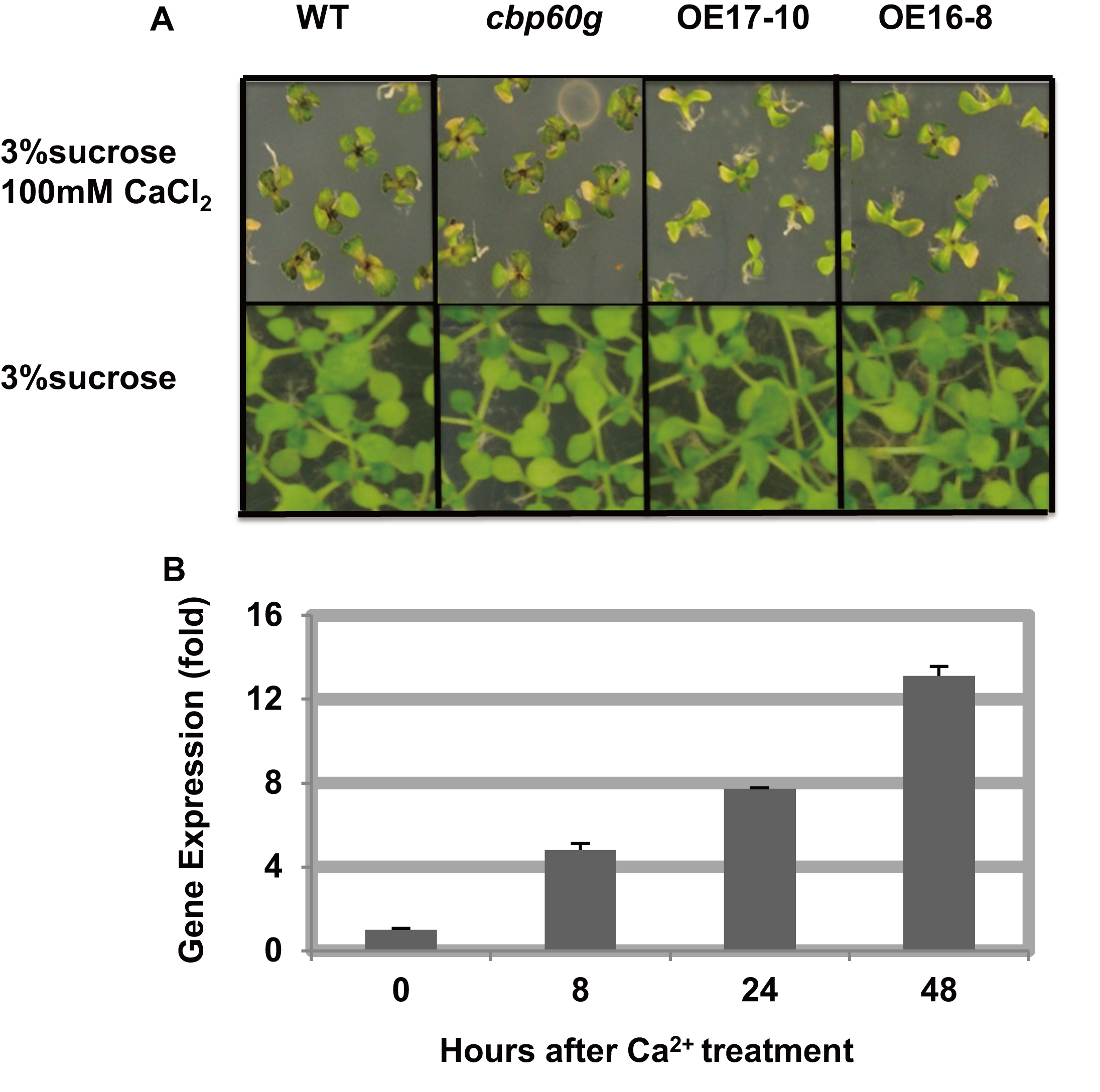

Supplement: S5 Fig — (A) Twelve-day-old wild-type, the cbp60g mutant and CBP60g overexpression lines grown in the solid 1/2 MS medium with 3% sucrose and 100 mM CaCl2 were observed. (B) The expression of CBP60g was induced under 50 mM CaCl2. (TIF) [file pone.0173129.s005.tif]

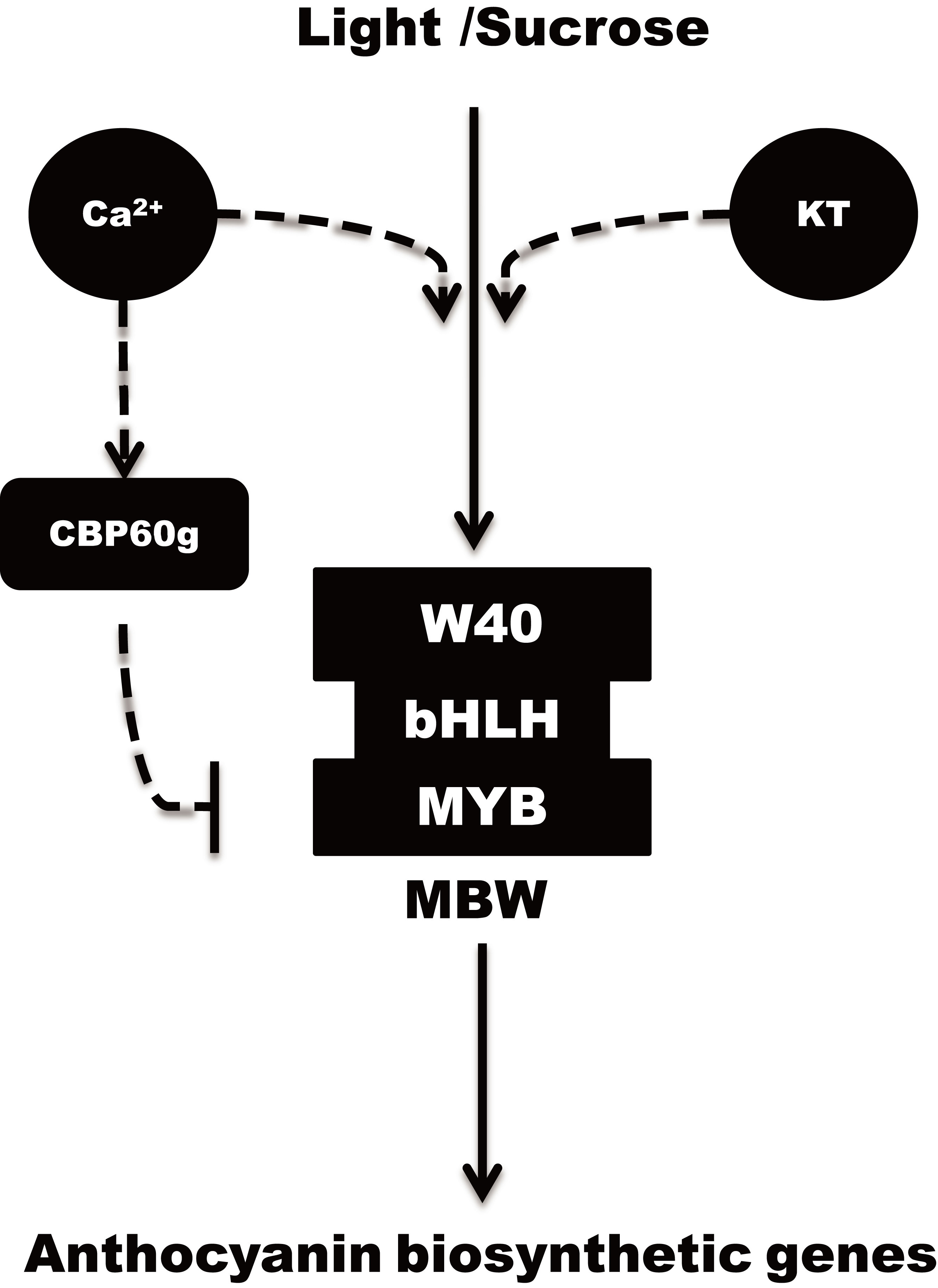

Supplement: S6 Fig — Light and sucrose induce anthocyanin accumulation, cytokinin and calcium signaling participate in this process. Calcium signaling increases the anthocyanin accumulation, while on the other hand, induces CBP60g expression thus represses the anthocyanin accumulation. These form a negative feedback. We suggest that CBP60g regulates anthocyanin accumulation through PAP1 and TT8, which are the components of MBW complex. (TIF) [file pone.0173129.s006.tif]
