# Supplementary material for: Calmodulin-binding protein CBP60g functions as a negative regulator in Arabidopsis anthocyanin accumulation
Source: PLoS One. 2017 Mar 2;12(3):e0173129. doi: 10.1371/journal.pone.0173129 (PMC5333885; doi:10.1371/journal.pone.0173129)
Supplement: S1 Table — (DOC) [file pone.0173129.s007.doc]

**Supplementary Table**

| Genes | Polarity | Primer sequences (5'to3') |
| --- | --- | --- |
|  |  |  |
| *EF1a* | F | AGAAGGGTGCCAAATGATGAG |
| *EF1a* | R | GGAGGGAGAGAGAAAGTCACAGA |
| *CBP60g* | F | AATAACGAGGAGGATGAGAACG |
| *CBP60g* | R | TCAGACACGGTAAGAAACATCG |
| *ARR5* | F | GGCTGAGGTTTTGCGTCCC |
| *ARR5* | R | TCCAGTCATCCCAGGCATAGAG |
| *CHS* | F | CTTCCCTCAAATGTCCGTCTATG |
| *CHS* | R | CGTGTCCTCGTTGTCTGCTCT |
| *CHI* | F | CCTTTTCGTCCTTGTTCTTCATCAT |
| *CHI* | R | GAGGCGGTTCTGGAATCTATCA |
| *PAP1* | F | CCATCTCAATGCCCCACCA |
| *PAP1* | R | TTCTGTTGTCGTCGCTTCAGG |
| *TT8* | F | GCAACAGCATAAGCGGACG |
| *TT8* | R | CCTCTTACTTTCGCCCTTATCTCC |
| *DFR* | F | AGGCGGCATAGACGTTGTGA |
| *DFR* | R | GGAGAAAGCAGCGTGGGATT |
| *MYBL2* | F | AGATTGCCAGGACGAACCG |
| *MYBL2* | R | GGAGACGATGATTGGTTGGGT |
| *TT2* | F | GATGGATCAAGGTGGATCTTCG |
| *TT2* | R | AAGTGAAGTCTCGGAGCCAATC |
| *PAP2* | F | ATAGGTGGTCCTTGATTGCTGG |
| *PAP2* | R | AGGATCGAGGTCGAGGCTTAA |
| *GL3* | F | TTTGCTTCCCGTTCCTTGG |
| *GL3* | R | AGGCTCCGTACTGAACATAGGC |
| *EGL1* | F | TTCAAGCGATTGCGTTTCCC |
| *EGL1* | R | GATGATCGCTTCCACCTTGTGA |

**Supplementary Table S1** Primers used for qRT-PCR analysis in this work. F, forward primer; R, reverse primer
